# Intra- and inter-species interactions in microbial communities

**DOI:** 10.3389/fmicb.2014.00629

**Published:** 2014-11-24

**Authors:** Luis R. Comolli

**Affiliations:** Beamline 4.2.2, Advanced Light Source, ALS-Molecular Biology ConsortiumBerkeley, CA, USA

**Keywords:** archaea, microbial communities, inter-species interactions, uncultivated biofilms, microbial networks

In this Special Topic we explore some of the novel mechanisms interconnecting microbes within and across species, and to their physical environment, across vastly different scales.

As developments in various “OMICs” fields have revolutionized our understanding of the vast diversity and ubiquity of microbes in the biosphere, we have also developed new holistic ways of thinking about them. Human microbiome scientists are currently thinking about the whole set of microorganisms in the human intestine as a single entity or as one organism (Li, 2014). In the confined environment of the human body and subjected to a tight interaction with the host, this conceptual shift from individual microbes and “species” to their integrated set of inputs and outputs may seem natural. Individual microbes react individually to the host environment in the context of other microbes and their mutual interactions, producing as a result an integrated collective behavior. The human body in turn processes and reacts to this aggregated result, the behavior and actions of the whole microbial community. Thus, while individual bacteria interactions occur at the nanoscale size range, bacterial communities are shaped by landscape structures from the microscale or larger and produce collective behavior at such a scale as well.

More open systems are potentially more complex, at least in terms of having variable or open physical boundaries and a less tightly regulated dynamic range of local properties. Nonetheless, across a wide range of diverse environments (soils, lakes, coral riffs, hot and acidic extreme environments, subsurface aquifers, and living organisms from plants to animals), whole populations of microorganisms have developed system-wide homeostatic adaptations to external factors (Fernandez et al., 2014 and references therein; Karatan and Watnick, 2009). The chemical transmissions of information underlying collective behaviors such as in quorum sensing have been recognized for a long time (Ryan and Dow, 2008 and references therein), but we are referring here to more intimate relationships. In the case of biofilms it is natural to compare them with tissues (Hall-Stoodley et al., 2004; Karatan and Watnick, 2009; Subbiahdoss et al., 2009), with various cell types and the extracellular substances as a matrix holding the whole together. Genomics data increasingly point toward the co-existence of metabolically incomplete individual “species” across environments, including microorganisms within planktonic systems such as subsurface aquifers (Wrighton et al., 2012; Castelle et al., 2013). There seems to be a wide range of phenomena beyond the use of chemical signals to synchronize behavior across entire populations.

The type and extent of microbe-microbe and microbe-host nutritional interactions will determine the metabolism of the entire community in a given environment. We would expect the choice between biofilm formation and planktonic growth to be accurately regulated. Indeed, Rajeev et al. (2014) report on two diguanylate cyclases (DGCs) in the bacterium *Desulfovibrio vulgaris* Hildenborough that function as part of two-component signaling pathway, each one specific for one choice of growth fate. Once the fate has been committed, the type of interactions, topological relationships and constraints, and the physical means to establish them determine metabolic strategies. Nutrient sharing and electron transfer among microbes are reviewed by Seth and Taga (2014), and Shrestha and Rotaru (2014), respectively. Microbial community members can also gather energy cooperatively, from chemical reactions no single species can catalyze. Two types of electron transfer between microorganisms are recognized: the transfer of chemical intermediate in redox reactions and direct electron transfer. These and possibly other modes of nutrient and energy sharing between microbes are just starting to be investigated through mechanistic and structural studies.

The physical means used by microbes to form networks or affect other microbes at a distance are surprising and somewhat counter-intuitive within old paradigms of species. Perras et al. (2014) examined uncultivated biofilms taken directly from a natural sulfidic marsh (Sippenauer Moor near Regensburg, Bavaria, Germany) by transmission and scanning electron microscopy (TEM and SEM). The dominant *SM1 Euryarchaeon* uses thin appendages to connect to other cells of the same species forming a network in which each cell has an average of six connections, but also connects to cells of other species. In fact, the archaeal cells appear to connect to bacteria, establishing an interaction across two kingdoms of life. Comolli and Banfield (2014) linked cryogenic TEM with genomics and proteomics to show a range of physical interactions and connections between archaeal cells of different species, including “synapse-like” and tubular connections through cell wall openings. The inner diameters of some of these connections are large enough to enable the exchange of the largest cytoplasmic macromolecules and molecular machines. Berleman et al. (2014) used conventional TEM, Mass Spectrometry analysis and biochemistry to investigate outer membrane vesicles (OMV) produced by *Myxococcus xanthus*, a bacterial micro-predator known for hunting other microbes. They analyzed the protein and small molecule cargo of OMVs conclusively proving that they are associated with antibiotic activity, including the product of gene *mepA*, an M36 protease homolog. Taken together these three contributions show physical means used by microbes to affect other microbes within their environment but at a distance: how they establish vast networks with new physical properties than those of individual microbes; how they interconnect across species physically, in principle enabling the exchange of gene products; and releasing enzymatic cargo.

Given a set of experimental observations, models allow us to explore the minimal set of rules and relationships that could account for the data; numerical simulations and models also serve to generate new hypothesis or extend questions beyond the available experimental data (Silva, 2011). In this Special topic Madeo et al. (2014) apply game theory in a first model that accounts for the observed patterns of inter-species interconnections in imaging data. Sinclair (2014) shows the counter-intuitive possibility of killer and prey co-existence, an insight of potentially wide impact. As more extensive imaging data across modalities shows us patterns and relationships for different types of microbial communities, and highly-resolved metabolomics capabilities resolving essential co-dependencies are developed, a new generation of modeling efforts of increasing power and sophistication will play a key role. New models will likely incorporate dozens to hundreds of secreted chemicals and metabolites that modulate the behavior, survival, and differentiation of members of the community, extending our ability to formulate new testable hypothesis.

We argue above that microbial communities in defined relationships or environments should be thought of holistically, and four papers in this Special Topic do just that. Holmes and co-authors investigated symbiotic associations of protozoa and endosymbiotic methanogens in groundwater communities (Holmes et al., 2014). They show how under certain conditions, the protozoa hosting endosymbionts become important members of the microbial community. As they feed on moribund biomass and produce methane, their system-wide conclusions are relevant for engineered bioremediation approaches in general. Hess and co-workers (Piao et al., 2014) used 16S rRNA gene profiling analysis of the cow cellulose-digesting anaerobic rumen ecosystem, where microbial-mediated fermentation degrades a complex mixture of cellulosic fibers. They show how diverse microbial taxonomic groups change in time, such that complete degradation is the results of their synergistic activity. Gathering an impressive dataset, Freilich and co-authors (Zchori-Fein et al., 2014) studied variations in the bacterial symbiotic communities of the sweet potato whitefly *Bemisia tabaci* (Hemiptera: Aleyrodidae). Compiling a dataset of over 2000 individuals derived from several independent screenings, the dataset is unprecedented in number of individuals as well as the geographical range and habitat diversity. Their work adds compelling evidence that facultative endosymbionts complement partial metabolic pathways in the host, thus modulating their distribution patterns. Guo et al. (2014) allow us to expand our framework from terrestrial microbiology to human oral microbial communities whose synergistic activities can be pathogenic to us. They surveyed evidence of cell contact-dependent physical interactions, metabolic interdependencies, and synchronizing signaling systems which are used to maintain a balanced microbial community but also induce pathogenic pathways if we do not control them. Mechanisms conferring robustness, adaptability, and integrated responses are present in microbial communities from habitats that may seem inhospitable to us, to microbial communities common in human environments.

Perhaps conceptual aspects of the “holobiont” play a role at an entirely more subtle level, as volatile-mediated interactions can be expected to play an important role in information sharing, synchronization, and competition among physically separated microbes. Garbeva et al. (2014) report the first experimental study indeed proving antibiotic production levels and gene expression changes in one bacterial species, *Pseudomonas fluorescens Pf0-1*, as a consequence of the exposure to volatiles produced by four different species. In these cases microbes are in direct contact, confined in a structured space, which they can alter to some degree, and to which they must adapt too. We can thus reason that these physical aspects inevitably lead to networks and interactions. However, in the case of microbes distributed in space at non-obligatory short distances, indeed long variable distances, the emergence of communications and networks through volatile chemicals seems to more forcefully challenge the idea of “single individuals” or “single species.” This work contributes to expanding how we think of the concept “holobiont.”

Microorganisms with unique positions in the kingdom of life and the complex web of interactions they participate in are of great interest, as they can show us either evolutionary remnants or novel ways to solve the same problems. Lage and Bondoso review the relatively understudied interactions between Planctomycetes and macroalgae in the context of complex microbial biofilms (Lage and Bondoso, 2014). Planctomcyetes share certain features with archaea, such as proteinaceous cell walls without peptidoglycan, and some distinctive characteristics with eukaryotes such as a complex system of endomembranes forming a unique cell plan. Completing our survey, Ounjai and Chaturongakul review how bacteriophages affect host gene expression (Chaturongakul and Ounjai, 2014). As microbes are under evolutionary pressure to improve environmental fitness, bacteriophages need to dynamically adapt to alter gene expression for their own survival. Microbes in turn can use part of the bacteriophages machinery as part of their tool box to compete with other microbes. They argue that imaging and structural work hold the key to further elucidating this complex evolutionary relationship.

We have presented a comprehensive survey showing physical interactions and connections between microbes of different species, co-occurrence patterns of distribution, and a range of metabolic interdependencies across environments. As stated in the opening of this Special Topic, we believe there is a compelling need for imaging data across modalities, providing physical characterizations linking metagenomics and metaproteomics to microbial patterns of distribution and networks. We also look forward for the development of novel imaging instrumentation and measurement technologies supporting an integrated analysis of communication among cytoplasmic compartments, between individual microbial cells, and within multicellular communities and biofilms.

## Conflict of interest statement

The author declares that the research was conducted in the absence of any commercial or financial relationships that could be construed as a potential conflict of interest.
